# Understanding cancer survivors’ reasons to medicate with cannabis: A qualitative study based on the theory of planned behavior

**DOI:** 10.1002/cam4.3536

**Published:** 2020-10-17

**Authors:** Helen McTaggart‐Cowan, Colene Bentley, Adam Raymakers, Rebecca Metcalfe, Philippa Hawley, Stuart Peacock

**Affiliations:** ^1^ Faculty of Health Sciences Simon Fraser University Canadian Centre for Applied Research in Cancer Control Cancer Control Research, BC Cancer Vancouver BC Canada; ^2^ Canadian Centre for Applied Research in Cancer Control Cancer Control Research, BC Cancer Vancouver BC Canada; ^3^ Cancer Control Research, BC Cancer School of Population and Public Health, University of British Columbia Vancouver BC Canada; ^4^ Pain & Symptom Management/Palliative Care Program BC Cancer Vancouver BC Canada

**Keywords:** Canada, cancer survivors, cannabis, decision making, qualitative, theory of planned behavior

## Abstract

**Background:**

Prior to nonmedical cannabis legalization in Canada, individuals were only able to access cannabis legally through licensed producers with medical authorization. Now with an additional legal access system designed for nonmedical purposes, it is unclear what factors influence cancer survivors’ decisions to medicate or not medicate cannabis as a complementary therapy to alleviate their cancer symptoms.

**Methods:**

We recruited cancer survivors via social media. Interested individuals were purposively sampled to ensure maximization in terms of age, sex, and province of residence. Constructs of the Theory of Planned Behavior were explored during the telephone interviews as participants described what influenced their decisions to medicate or not medicate cannabis to manage their symptoms.

**Results:**

Interviews were conducted with 33 cancer survivors. All individuals believed that cannabis would manage their cancer symptoms. Those that chose to medicate with cannabis provided a variety of reasons, including that cannabis was a more natural alternative; that it reduced their overall number of prescription drugs; and that safer products had become available with the legalization of nonmedical cannabis. Some individuals also indicated that support from physicians and validation from family and friends were important in their decision to medicate with cannabis. Individuals who opted not to medicate with cannabis raised concerns about the lack of scientific evidence and/or possible dependency issues. Some also felt their physician's disapproval was a barrier to considering cannabis use.

**Conclusions:**

The findings revealed that recreational legalization made using cannabis appear safer and easier to access for some cancer survivors. However, physicians’ censure of cannabis use for symptom management was a barrier for survivors considering its use.

## Graphical abstract

This manuscript illustrates the findings from individual interviews conducted with Canadian cancer survivors to explore their reasons to use cannabis as a complementary therapy to manage their cancer symptoms. In Canada, we have a unique opportunity to study the impact of country‐wide recreational legalization of access to non‐medical cannabis on cancer survivors’ attitudes toward use of those substances. We used the theory of planned behaviour as the decision‐making model to understand intention and behaviour of cancer survivors regarding cannabis. The findings from our work will better inform healthcare providers how to support their patients regarding cannabis medication and to promote person‐centred and informed decision‐making.

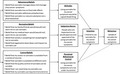

## 1 INTRODUCTION

Cancer survivors––individuals diagnosed with cancer until the end of their life^1^––have reported medicating with cannabis or drugs containing cannabinoid.^2^ They report favorable outcomes for managing chemotherapy‐induced nausea and vomiting, cancer‐related pain, anorexia, insomnia, and depression,^3^, ^4^ as well as an improvement in their quality of life.^5^ While there are a limited number of high‐quality clinical trials evaluating the effectiveness of cannabis in alleviating cancer symptoms,^6^, ^7^ some countries have legalized nonmedical cannabis. In Canada, the Cannabis Act^8^ (effective as of 17 October 2018), which replaced the Access to Cannabis for Medical Purposes Regulations,^8^ enables individuals to purchase cannabis and cannabinoid products through licensed producers for personal use without medical documentation.

The Canadian Medical Association (CMA) has recognized the unique requirements of individuals suffering from chronic diseases––including cancer––for which conventional therapies have not been effective and for whom cannabis may provide relief.^9^, ^10^ However, the CMA raises concerns about the lack of evidence on the risks and benefits associated with the use of cannabis. The CMA believes that physicians should not be gatekeepers because cannabis has not undergone the established regulatory review processes that are required for all prescription medicines. Oncology health‐care providers reported that they lacked sufficient cannabis knowledge to make recommendations to their patients.^11^ The lack of readily accessible information in standard medical textbooks regarding dosing requirements and potential side‐effects of cannabis use may make it challenging for physicians when advising their patients.

The recent full legalization of nonmedical cannabis in Canada may result in cancer survivors opting to medicate their cancer symptoms without guidance from health‐care providers.^12^ However, there have been limited studies determining the frequency of cannabis use among cancer patients in Canada.^13^, ^14^, ^15^, ^16^ A 2017 survey of cancer patients in the province of Alberta revealed that 43% had tried cannabis.^15^ In British Columbia (BC), a 2018 cross‐sectional study showed that 23% of cancer patients were currently using cannabis‐based products to manage their symptoms or treat their cancer, or both; 31% of these individuals had medical authorization.^16^ These few studies further contribute to the problem of insufficient evidence regarding the risks and benefits of medicating with cannabis.

It is vital to better inform health‐care providers to support their patients regarding cannabis medication and to promote person‐centered and informed decision making. In Canada, we have a unique opportunity to study the impact of country‐wide recreational legalization of access to nonmedical cannabis on cancer survivors’ attitudes toward use of those substances. The aim of the current interview study was to gain an understanding of the factors that influence cancer survivors’ decisions to medicate or not medicate with cannabis as a complementary therapy to alleviate their cancer symptoms.

## 2 METHODS

### 2.1 Recruitment

Canadian cancer survivors were recruited using social media to participate in this interview study by first completing an online eligibility survey to express their willingness to participate in the interview. The study was advertised on Twitter and Facebook using our investigators’ channels, as well as several cancer organizations (e.g., Canadian Centre for Applied Research in Cancer Control, BC Cancer) that supported our recruitment efforts. For the purpose of this study, we used the National Cancer Institute's (NCI) definition of a cancer survivor, which is an individual from the time of diagnosis until the end of life.^17^ The rationale for using the NCI’s definition was to capture individuals’ experiences across the cancer care continuum. In the eligibility survey, individuals provided information about their sex, age, province/territory of residence, month/year of cancer diagnosis, type of cancer, and whether or not cannabis was used to medicate cancer symptoms or treatment side‐effects. The recruitment period was from September 2019 to November 2019. The study protocol was approved by the BC Cancer Research Ethics Board (H19‐01489).

Of the 115 individuals who opened the eligibility survey, 111 provided complete responses. The research team purposively sampled individuals to ensure maximization with respect to age, sex, province/territory of residence, and whether or not cannabis was used to control or alleviate cancer symptoms or treatment side‐effects. Of the 111 individuals who consented to be contacted to participate in the study, 46 individuals were sent an invitation email to schedule an interview time. Participants received an honorarium of $50 (CAD).

### 2.2 The Theory of Planned Behavior and the interview process

**FIGURE 1:**
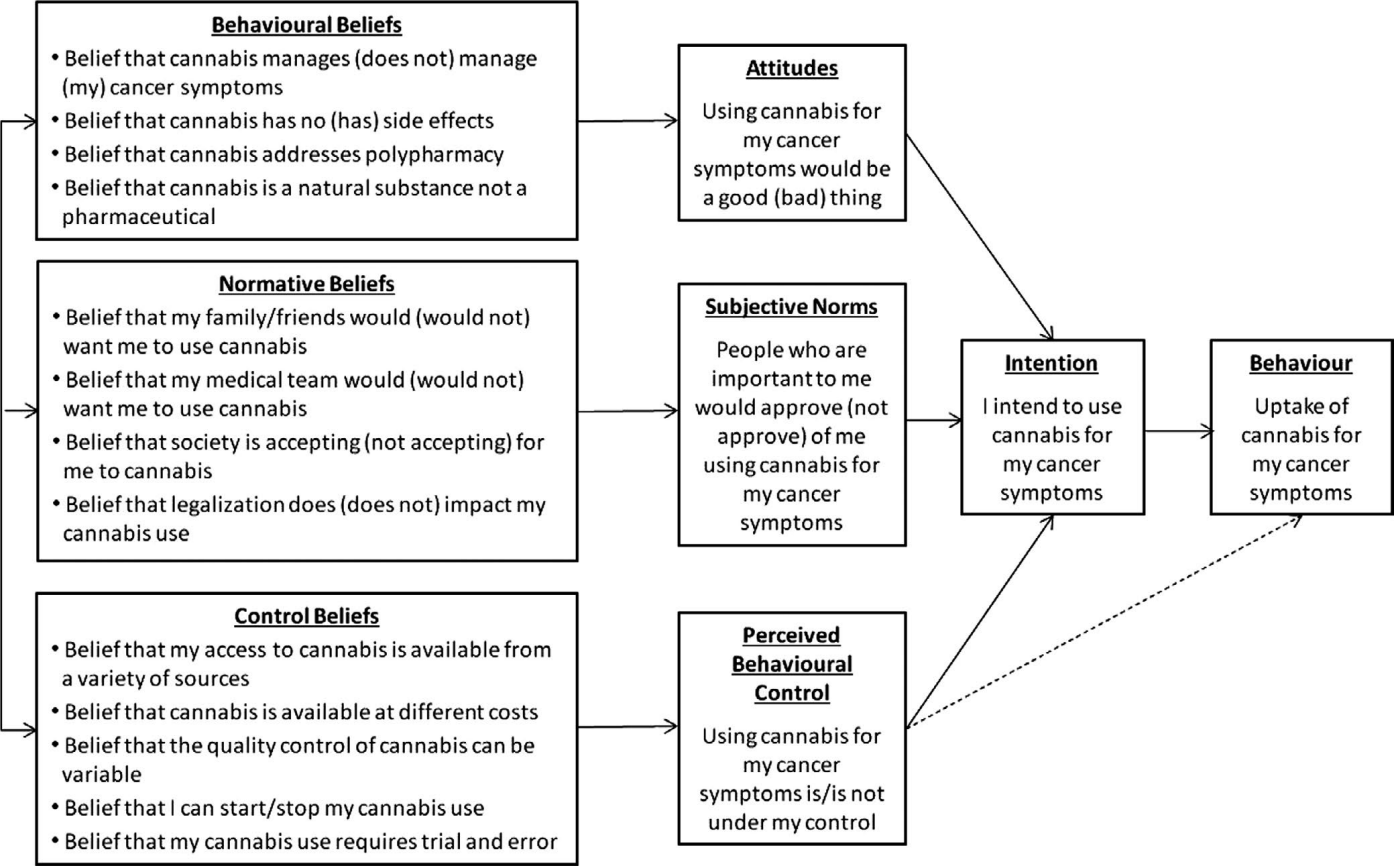
Schematic of theory of planned behaviour for individuals’ decision to medicate with cannabis

An interview guide (available as an electronic Supplementary material) was developed informed by the Theory of Planned Behavior (TPB). Participants were asked to describe what influenced their decisions to manage their cancer symptoms with cannabis (Figure 1). The TPB is a well‐validated, psychological, theory‐based, decision‐making model that proposes three constructs which together predict an individual's intention to perform a specific behavior.^18^ These constructs are attitudes (i.e., how positively (or negatively) a behavior is appraised); subjective norms (i.e., perceived social pressure to perform (or not perform) a behavior); and perceived behavioral control (i.e., perceived ease (or difficulty) of performing a behavior). Influencing each of the three constructs are the associated underlying beliefs. Specifically, behavioral beliefs (i.e., advantages and disadvantages of performing the behavior) influence attitudes; normative beliefs (i.e., whether specific individuals approve or disapprove) underlie subjective norm; and control beliefs (i.e., barriers and facilitators) influence perceived behavioral control.

The interview questions were open‐ended and probes were used when it was necessary to clarify responses or to gain more information. All interviews were conducted over the telephone by a trained interviewer (RM), and lasted between 14‐60 minutes (average: 30 min). The interviews were digitally recorded and transcribed by a third party.

### 2.3 Data analysis

The transcripts were de‐identified, and analyzed using the initial codes based on the TPB structure and organized in NVivo software (QSR International Pty Ltd., Version 12, 2019). A main qualitative analyst (HMC) read and coded all transcripts to get a comprehensive sense of the data, a second analyst (CB) independently read and coded approximately half of the transcripts (52%), and a third analyst (AR) read 30% of the transcripts. Weekly norming sessions were held with the three analysts and the interviewer. The purpose of these sessions was to resolve disagreements in the interpretation and coding of transcripts by group discussion and consensus; iteratively revise understandings of the data; and determine when new categories or concepts could be derived from the data. The main analyst kept an audit trail of coding decisions and meetings. The findings were stratified by participants reporting to medicate their cancer symptoms with cannabis (MWC) and those reporting that they do not medicate with cannabis (nMWC). By presenting stratified findings, a deeper understanding of the factors that influence medicating with cannabis could be gained.

## 3 RESULTS

### 3.1 Study participants

Of the 46 individuals invited to the study, 33 cancer survivors participated (Table 1). The majority of the study participants were female (61%) and resided in either Ontario (30%) or BC (27%). The sample consisted of slightly more MWCs (52%) than nMWCs. The 13 individuals, who did not respond to our email invitation, did not provide reasons for declining participation. The majority of these individuals were female (85%), under the age of 40 years (62%), and were MWCs (69%).

**TABLE 1:** Characteristics of the study sample

	Count (%)
Sex	
Male	13 (39%)
Female	20 (61%)
Current age (years)	
<30	5 (15%)
30‐44	9 (27%)
45‐64	10 (30%)
>65	9 (27%)
Province of residence	
British Columbia	9 (27%)
Alberta	6 (18%)
Ontario	10 (30%)
Quebec	2 (6%)
New Brunswick	1 (3%)
Nova Scotia	4 (12%)
Newfoundland	1 (3%)
Medicating cancer symptoms with cannabis	
Yes	17 (52%)
No	16 (48%)
Most recent cancer diagnosis	
Colorectal	6 (18%)
Breast	6 (18%)
Ovarian	5 (15%)
Lymphoma	3 (9%)
Leukemia	2 (6%)
Prostate	2 (6%)
Thyroid	2 (6%)
Gastric	1 (3%)
Germ	1 (3%)
Lung	1 (3%)
Multiple myeloma	1 (3%)
Neck	1 (3%)
Testicular	1 (3%)
Tongue	1 (3%)

### 3.2 Findings from the interviews

In terms of behavioral beliefs, all participants believed that medicating with cannabis could help to manage cancer symptoms: “it was good for […] nausea; it was good for sleeping; it was good for depression, anxiety [;…] it was good for joint pain as well” (ID27, MWC). MWCs indicated they opted to use cannabis to address issues of polypharmacy, such that cannabis could replace one or many of their prescribed medications. While nMWCs perceived that cannabis had benefits to alleviating cancer symptoms, they firmly believed that medicating with cannabis would lead to undesired side‐effects and possibly interact with their currently prescribed medications. For normative beliefs, family and friends were more likely than the medical community to approve of cannabis use. For control beliefs, both MWCs and nMWCs held the perceptions that easily accessible cannabis facilitates cannabis use. The most reported barrier among nMWCs was concern about possible dependency issues.

More detailed findings from each of the TPB constructs are described below.

#### 3.2.1 Behavioral beliefs

Not all participants made the decision to medicate with cannabis. For some nMWCs, the lack of cannabis information, including availability of different products (e.g., edibles), led them to perceive that the side‐effects from taking cannabis were undesirable.

[…] I don't like anything in my lungs […]. And then the other reason was that I heard that people had the munchies with it and I didn't want to keep eating because of [the cannabis]. (ID24, nMWC).

Some nMWCs stated that they felt cannabis was not necessary as an initial approach to alleviate their cancer symptoms. One participant stated that she “[…] always had medications to turn to or other techniques” and recognized that she “[…] had a really complicated course of treatment with a lot of unusual side‐effects and drug interactions [… and…] didn't think that adding an unknown [cannabis…] was a good idea […]” (ID2, nMWC).

Alternatively, participants who medicated with cannabis did not perceive cannabis to have significant side‐effects on their health. A desire was observed among some MWCs to medicate their cancer symptoms with a “natural” substance “that grows [in the] wild, [as] it did for centuries” (ID7, MWC). There was a perception among some MWCs that cannabis can address issues of polypharmacy.

I actually refrained from using some of my chemotherapy pills in exchange for marijuana, like the nausea pills. (ID27, MWC).

#### 3.2.2 Normative beliefs

Both MWC and nMWC participants reported a variety of individuals in their lives whose opinions were important to them regarding their decision to medicate their cancer symptoms with cannabis. The opinions held by these individuals affected the participants by: (a) influencing the level of support they received and (b) influencing their willingness to be open about medicating, or to consider medicating, with cannabis.

*Family and friends*. Many MWC and nMWC participants valued the opinions of family members and friends, even if their opinions were misinformed. Participants medicating with cannabis reported that family and friends were generally supportive of this practice. They reported that their family and friends held the hope that the cannabis “will help cure [… or provide] relief of [their cancer] symptoms” (ID13, MWC).

It was apparent that participants desired the approval from family and friends about medicating with cannabis. Different approaches were observed among participants with how they sought approval. One nMWC participant, for example, stated she would be open to her family members if she were to consider cannabis for her cancer symptoms.

[…] I also am concerned, always, with what my family thinks and their sort of approval of me. So, I think I was just worried because they know that […] I’ve never tried cannabis, so I think maybe they may be concerned, like "Why? Why are you doing this now?”. (ID4, nMWC).

Moreover, there were MWCs and nMWCs with strong desires to gain the approval of their family that they made the decision to not fully disclose that they are medicating, or considering medicating, with cannabis. These participants had an underlying concern of their family's disapproval of cannabis.

[…] I kept [my medicating with cannabis] a secret […] because I have children and I’m not too sure how they would react.

(ID6, MWC).

[…B]etween you and me, probably wouldn't mention [cannabis, if I decide to medicate with it] initially to my husband. (ID24, nMWC).

*Medical team*. Both MWCs and nMWCs valued the opinions of their medical team when making the decision to medicate cancer symptoms. They believed that their physicians “[…] know exactly what's happening [when…] they say that this drug works and that drug doesn't work […]” (ID15, nMWC). Because of this view, many nMWCs preferred that their medical team provided clinical guidance on medicating with cannabis.

If [medicating with cannabis] was recommended to me or prescribed to me by my oncologist or anyone on her team then, you know, that to me would say they absolutely support its use and see that there is high value. The fact that they don't even raise the subject, you know, says to me they may have some concerns about the value. (ID9, nMWC).

MWCs described different levels of approval their medical team had in regards to medicating with cannabis. Some physicians were agreeable to a prescription for cannabis or a referral to a medical cannabis specialist but others were not. One participant highlighted the challenge with accessing cannabis even though their medical team was supportive of cannabis.

The family doctor that I have is highly supportive of whatever works for me and makes me comfortable considering the circumstances. However, did not prescribe [cannabis], but she was very supportive but couldn't facilitate it, fine. Cancer doctor was also very open and very supportive [of medicating cancer symptoms with cannabis] but also […] doesn't deal with any of the prescription […]. (ID16, MWC)

Not all MWCs were willing to fully disclose that they were medicating with cannabis to their medical team because they recognized that their doctor would disapprove. One participant said that his “[medical team…] would never recommend that I do [cannabis…]. They don't like that I do it, so but they don't tell me not to either” (ID27, MWC). Another participant described the consequence of his doctor ever learning of his cannabis use.

My own personal physician that I've had for 25 years said, “If you're using [cannabis] then you'd better find a new doctor because I won't see you anymore.” So I've had to hide it from him because otherwise he's kicking me out the door. (ID14, MWC)

Both MWCs and nMWCs expressed that the medical team's disapproval also resulted in communication challenges if they wanted to have a discussion about cannabis at their appointments.

[…W]hen I ask [… my doctor] about cannabis or anything she […] just put[s] her hands up in the air and stops me, and goes like “We can't talk about this.” So, literally there's a physical block of being able to talk about it, so that's weird to me. (ID13, MWC)

[…] I went to my family doctor and […] I said, “What about cannabis?” And she said, “Oh, you don't want to get into that. That just creates more issues for you.” So she ditched it, so it was dead in its tracks. (ID24, nMWC).

*Society*. Canada's legalization of nonmedical cannabis elicited mixed responses among the participants regardless of whether they medicated with cannabis or not. Some MWCs and nMWCs felt that cannabis should have always been legalized. However, for some MWCs and nMWCs, the legalization removed the stigma of using cannabis.

[The legalization of cannabis] gives me a chance to remove the illegal, […] you could go to jail stigma attached to it. […] I just don't want to do something that the law says is inappropriate to do in our society. So the fact that cannabis has been legalized gives me an opportunity to feel comfortable about using it […]. (ID24, nMWC).

For some participants, legalization had little impact on their decision‐making process. For MWCs, they made the decision to medicate with cannabis before legalization. For some nMWCs, legalization did not change their willingness to medicate with cannabis due to their work obligations (i.e., international travel). One participant stated that “just because [cannabis is] legal doesn't mean that it's something for everyone at all times” (ID12, nMWC).

#### 3.2.3 Perceived behavioral control

A range of facilitators and barriers to medicating with cannabis was reported. The different modes of access were the most commonly reported facilitator to medicating with cannabis. Legalization has improved the accessibility of cannabis for some participants, who discussed the ease in accessing cannabis without “the hassle of like going through all that process [of obtaining a medical cannabis document]” (ID29, nMWC).

[…] I think [the legalization of cannabis] just made it more convenient, frankly, because it's a shit show trying to get your [cannabinoid] products through the medicinal sometimes […]. So it's the convenience to go down to the corner and grab pre‐rolls, or flower, or oil, whatever you need, and now you'll be able to get edibles too. (ID16, MWC).

The legalization of cannabis elicited a range of perceptions from the participants regarding the safety of the product they were using. Some expressed that there was now no need “to worry about [the cannabis’] quality” (ID18, MWC). Some participants had no concerns about the safety of the cannabis product before legalization.

I'll be frank with you; I don't buy it from a legal store […]. I have a private source. […T]his individual makes other things from the weed that he buys. And the quality of the other products that he makes is very good […]. (ID7, MWC).

MWC participants expressed that cannabis products are available at different costs depending on how you choose to access them. One participant stated that she has kept her medicinal cannabis designation “because [my cannabis is] tax deductible and I've been approved for tax credits […]” (ID16, MWC). For many MWCs, they stated their preference was still to access cannabis from their private sources as they did before legalization because they can purchase it for “a lot less than what those stores are asking for” (ID7, MWC).

Participants needed to feel comfortable with experimenting with cannabis to determine the right product and required dosage to help with their symptoms. One participant knew she was “[…] perfectly confident that you know your own body, you know when you felt good and you know you're having a reaction […]” (ID1, MWC). However, another participant expressed hesitation about the amount of cannabis she was prescribed to use.

[…R]ight now I'm taking maybe like a lower amount of what the physician has prescribed because I'm waiting to gather some more information and before starting what he recommended […]. (ID21, MWC)

Participants had mixed perceptions about the ability to start and stop medicating with cannabis. nMWCs raised concerns about “dependency issues [… and did not want to] become heavily dependent on [cannabis…]” (ID2, nMWC). MWCs stated that if they were asked to stop using cannabis that they were able to; however, only a few participants reported demonstrated that they were able to stop medicating cannabis on certain occasions.

[…] I went on a couple international trips, so of course I didn't travel with any cannabis, and I had no issues sleeping without it. (ID8, MWC).

## 4 DISCUSSION

Guided by the TPB and a strong sample size, this study gained an understanding of the motivations of Canadian cancer survivors who made the decision to medicate or not medicate their cancer symptoms with cannabis. Overall, study participants generally reported favorable attitudes toward medicating their cancer symptoms, such as pain and insomnia, with cannabis; this finding has been reported elsewhere.^16^ Despite these perceived benefits, not all participants made the decision to manage their cancer‐related health needs with cannabis.

The cancer survivors in the study were affected differently by their behavioral, normative, and control beliefs regarding medicating with cannabis. The importance of the constructs in predicting behavior appeared to be impacted by the level of experience the individual has had with cannabis. nMWCs wanted more information to address their concerns about unwanted side‐effects and potential interactions with medications they are currently taking. Conversely, MWCs typically required no additional information because they already had strong perceptions of the benefits of cannabis, which included reduction of burden related to polypharmacy; this finding has also been reported elsewhere.^16^ Cost was a concern for some MWCs but due to their experience, they were aware of the availability of options to purchase cannabis; this, in turn, provided them with a sense of control over how much they spent on cannabis.

The legalization of nonmedical cannabis in Canada played a role for some participants in their decision‐making process. Legalization provided them with a sense of social acceptance and legitimacy for them to medicate with cannabis. This was reflected in their discussions of wanting to portray a desirable image of themselves, as well as to others. Furthermore, legalization led them to feel that the quality control of cannabis had improved compared to products prior to legalization (i.e., consumed products have accurate labeling and do not contain molds or pesticides^16^). For individuals who had previously been medicating with cannabis long term, legalization had no impact. These individuals still preferred to obtain their cannabis from their private and unregulated sources at lower costs.

The normative beliefs held by individuals appear to have the greatest impact on whether they made the decision to medicate their cancer symptoms with cannabis. It was apparent that many participants desired the approval of important individuals in their lives, especially their medical team, when making this decision. A mixed level of physician comfort discussing cannabis was observed. Some participants reported that their physicians were willing to facilitate access to cannabis by writing a prescription or providing a referral to a medical cannabis expert. Moreover, some participants reported frustration and dissatisfaction with their physicians’ lack of willingness to discuss cannabis as an option for managing their cancer symptoms; this potentially could have led some participants to be misinformed about medicating with cannabis (i.e., cannabis curing cancer).^19^ However, some participants reported medicating with cannabis, despite not getting recommendations from their physicians, as their behavioral and control beliefs were stronger than their normative beliefs.

Physicians’ willingness to recommend cannabis to their patients may be dependent upon the policies set in place by their respective medical regulatory authorities (i.e., professional colleges). Many Colleges prohibit or strongly discourage dispensing, providing, or accepting delivery of cannabis for medical purposes, which likely impacts physicians’ willingness to recommend cannabis for their patients. In addition, a recent study reported that oncology health‐care providers felt that they lacked the knowledge to recommend cannabis to their patients because they desired the ability to monitor their patients’ cannabis use and to prescribe accurate doses^11^; as a result, physicians did not feel ready, and did not want to, answer patients’ questions about medical cannabis.^20^, ^21^

To the best of our knowledge, this is the first study that applied the TPB to understand the impact of national legalization of cannabis on cancer survivors’ decision to medicate or not medicate with cannabis using qualitative methodology. While Victorson et al. explored attitudes of American cancer survivors regarding medical cannabis for their symptom management using focus groups,^5^ results from the United States (US) are difficult to interpret, as state‐level legalization legislation remains in conflict with federal law.^22^ Thus, the legal status of cannabis remains ambiguous, and patients’ attitudes in the United States will be influenced by this ambiguity. That said, several of our findings were similar to those reported by Victorson et al. Similar findings included benefits for symptom management and side‐effect relief, concerns regarding the social stigma with the image of using cannabis among their social circles, cost of cannabis, and worry about the legal consequences were raised; though for our study, the last point is now alleviated with the Canadian legalization of nonmedical cannabis. While the burden related to polypharmacy was not raised, Victorson et al. found that their study sample believed that cannabis was a safer alternative to many conventional medications that they were prescribed.

Individuals with a range of tumor sites participated in the study; the most prevalent were colorectal and breast cancers, which aligned with the most recent Canadian cancer statistics.^23^ Lung and prostate cancers were the other most prevalent cancers in Canada but individuals diagnosed with lung and prostate cancers were under‐represented in our study. Using social media to recruit study participants allowed us to gain a wide range of perspectives from cancer survivors in Canada. The participants, however, were primarily recruited from Ontario and BC, the two most populous provinces. While social media broadened the recruitment coverage, we may have missed perspectives from individuals who do not have Internet access or do not follow social media. Given the online nature of our recruitment strategy, we relied on convenience sampling, which may lead to noncoverage bias in that the decision to participate in the study is at the discretion of the individuals and we do not have means of knowing about those who do not participate.^24^ As all interviews were conducted in English, we may have missed out on important viewpoints of non‐English speaking cancer survivors. We were able to gain nearly equal perspectives of MWCs and nMWCs to ensure that conversations were not highly in favor of medicating cancer symptoms with cannabis. In addition, our analysis highlighted the complexities that were faced by nMWC participants, who were considering cannabis for their cancer symptoms.

This study has shown that individuals affected by cancer positively perceived cannabis to alleviate symptoms such as pain, neuropathy, and insomnia. Our data revealed that, despite this favorable attitude toward cannabis, not everyone made the decision to medicate with cannabis. Individuals’ behavioral, normative, and control beliefs regarding medicating with cannabis provided vital information on what motivated their uptake of cannabis. An investigation of the underlying beliefs regarding medicating with cannabis can provide information on what motivates this behavior and may offer important future research avenues, including optimizing follow‐up cancer care. This information is important to health‐care providers in cancer as they receive the professional training and formal education required to support and educate cancer patients and survivors to improve self‐care, knowledge, and overall empowerment.

## CONFLICT OF INTEREST

PH will be receiving free study products from a licensed producer (Whistler Medical Marijuana Co, now owned by Aurora) for a clinical trial for which she is the Principal Investigator; she will not receive any financial benefits for conducting the study. SP is a member of the Board of Directors of the Canadian Agency for Drugs and Technologies in Health.

## AUTHOR CONTRIBUTIONS

HMC designed the study, analyzed the data, and prepared the first draft of the manuscript. CB and AR contributed to the study design and data analysis. RM conducted the interviews and contributed to data analysis. PH and SP contributed to the study design. All authors reviewed and approved the final version of the manuscript.

## Supporting information

Supplementary Material

## Data Availability

Data are available from the corresponding author upon reasonable request.
