# Supplementary material for: Understanding cancer survivors’ reasons to medicate with cannabis: A qualitative study based on the theory of planned behavior
Source: Cancer Med. 2020 Oct 17;10(1):396–404. doi: 10.1002/cam4.3536 (PMC7826491; doi:10.1002/cam4.3536)
Supplement: Supplementary file 1 — Supplementary Material [file CAM4-10-396-s001.docx]

**Cancer Survivors’ attitudes toward cannabis use topic guide**

Part 1: This is Part 1 of the interview, where I will ask you a couple of questions about your thoughts about cannabis and its ability to manage control or reduce your cancer symptoms or side effects.

1. [ALL PARTICIPANTS] Do you feel that cannabis can improve an individual’s cancer symptoms or side effects?
2. [ALL PARTICIPANTS] During the time you’ve been a cancer survivor, have you used cannabis to manage or reduce your cancer symptoms or side effects?
   1. [PARTICIPANTS ANSWERING YES TO Q2, ASK] What symptoms/side effects were you trying to manage/reduce?
   2. [PARTICIPANTS ANSWERING YES TO Q2, ASK] Do you feel that using cannabis has helped with your symptoms/side effects?
   3. [PARTICIPANTS ANSWERING NO TO Q2, GO TO Q3]
3. [ONLY FOR PARTICIPANTS ANSWERING NO TO Q2]: During the time you’ve been a cancer survivor, have you ever considered using cannabis to manage or reduce your cancer symptoms or side effects?
   1. [PARTICIPANTS ANSWERING YES TO Q3, ASK] For what symptoms/side effects would you consider using cannabis to manage/reduce?
   2. [PARTICIPANTS ANSWERING YES TO Q3, ASK] Why do you feel using cannabis will help with your symptoms/side effects?
   3. [PARTICIPANTS ANSWERING NO TO Q3, GO TO PART 2]

Part 2: This is Part 2 of the interview, where I will ask you to describe your reasons for using or not using cannabis during the time of being a cancer survivor.

1. [PARTICIPANTS ANSWERING YES TO Q2 AND Q3, ASK] Has your willingness to use cannabis/willingness to consider using cannabis to manage or reduce your cancer symptoms/side effect increased in the past few months? Why?
   1. Change in personal perception of cannabis (use)?
   2. Change in perception of cannabis (use) by family/friends? Why is this change in perception important to you?
   3. Change in perception of cannabis (use) by your doctor? Why is this change in perception important to you?
   4. Change in perception of cannabis (use) by your survivorship committee? Why is this change in perception important to you?
   5. Canadian legalization of cannabis? Why?
   6. Ease of cannabis access? Why?
      1. Accessibility of retail outlets
   7. Ease of cannabis delivery? Why?
      1. Smoking? Oral?
   8. Better safety profile of cannabis after legalization? Why?
      1. Better concentrations of THC and CBD?
2. [PARTICIPANTS ANSWERING NO TO Q2 AND Q3, ASK] You mentioned that as a cancer survivor, you have not used/considered using cannabis for your symptoms/side effects. Is there a particular reason why you haven’t? Please explain.
   1. Concern that it goes against my personal values/principles?
   2. Concern about what my family/friends may say?
   3. Concern about what the survivorship may say?
   4. Concern about not being prescribed by my doctor/oncologist?
   5. Concern about the side effects of cannabis? (e.g., second hand smoke, odour)
   6. Concern about my out-of-pocket cost?
   7. Concern of not being able to manage my cannabis use (in the long term)?
3. [ALL PARTICIPANTS] Has the October 2018 Canadian legalization of cannabis played role in whether or not you use cannabis? Please explain.

Part 3: And finally, for Part 3, I will ask you for your thoughts about managing your cannabis use.

1. [PARTICIPANTS ANSWERING YES TO Q2 AND Q3, ASK] If you decided to change how you use cannabis – for instance, if you wanted to stop using it or use it less often – would it be easy for you to make that change? Please explain.
   1. What factors will facilitate your change in cannabis use?
   2. What factors will inhibit your change in cannabis use?

[ALL PARTICIPANTS] This concludes the interview. But before I turn the audio recorder off, is there anything about cannabis use that I haven’t covered and that you’d like to talk about?

1. Is there something about cannabis use and cancer survivorship that is important to you that you want to share?

Thank you for your time.
